# Aptamer-Coated PLGA Nanoparticles Selectively Internalize into Epithelial Ovarian Cancer Cells In Vitro and In Vivo

**DOI:** 10.3390/biom15081123

**Published:** 2025-08-04

**Authors:** Gregory Benedetto, Anthony Fowler, Dan Langdon, Maya Raine, Molly Lynne White, Joshua Ogle, Corey Garmon, Craig Ogle, Christine Richardson

**Affiliations:** Center for Biomedical Engineering and Sciences, University of North Carolina at Charlotte, 9201 University Blvd., Charlotte, NC 28223, USA; gbenedetto4501@gmail.com (G.B.); anthony.fowler@sanofi.com (A.F.); dlangdo2@charlotte.edu (D.L.); mraine@charlotte.edu (M.R.); mwhit160@charlotte.edu (M.L.W.); joshua.ogle@hfsinclair.com (J.O.); n4mg@bpt.eurofinsus.com (C.G.); cogle@uncc.edu (C.O.)

**Keywords:** aptamer, targeted therapy, functionalized nanoparticle, personalized medicine, xenograft model epithelial ovarian cancer

## Abstract

Ovarian cancer is a deadly gynecological malignancy that will affect about 21,000 women and result in almost 153,000 deaths in the United States in 2025. New clinical tools that facilitate early diagnosis and treatment of ovarian malignancies will significantly help reduce mortality and improve current long-term survival rates. We utilized a previously identified single-strand DNA aptamer RLA01 that binds and internalizes into target epithelial ovarian cancer cells to label PLGA-based nanoparticles and determine their ability to selectively target EOC cells and deliver payloads for cellular internalization. Nanoparticles labeled with RLA01 significantly enhanced cellular uptake 20–85% by receptor-mediated endocytosis into target EOC Caov-3 cells and inhibited cellular uptake in non-target HOSE 6-3 cells. Further, labeling of paclitaxel-loaded nanoparticles with RLA01 significantly decreased cell proliferation and induced apoptosis. A preliminary pilot study looking at the in vivo stability of aptamers demonstrated their ability to promote retention and honing of nanoparticles at tumors. These data demonstrate the effective combinatorial use of aptamer RLA01 and nanoparticle technologies for the direct targeting of tumor cell populations both in vitro and in vivo.

## 1. Introduction

Ovarian cancer is a deadly gynecological malignancy that will affect about 21,000 women and result in almost 153,000 deaths in the United States in 2025 [1]. A woman’s chance of being diagnosed with invasive ovarian cancer in their lifetime is 1 in 72, and although ovarian cancer represents 1.3% of all newly diagnosed cancers nationally, it is the fifth leading cause of cancer-related deaths among women [1]. The most common malignancy of the female reproductive tract is epithelial ovarian cancer (EOC), which constitutes 75% of all new ovarian-related cases diagnosed each year [2]. Localized stage I EOC has a 92% 5-year survival rate but is only diagnosed in 15% of new cases due to the asymptomatic nature of EOCs [1,3]. The majority of diagnosed EOCs are stage III or IV (>75%) and carry a poor long-term prognosis (10–30%, 5-year survival) [4]. Chemotherapy and surgical resection is the primary treatment for ovarian cancer, but the pitfall of chemotherapy protocols is they are limited due to associated adverse systemic effects and high risk of recurrence, resulting in chemo-resistance [4,5].

Platinum taxanes that disrupt mitotic division and inhibit DNA repair, such as cisplatin, carboplatin, and paclitaxel, are front-line therapeutics used to treat ovarian tumors [5]. In addition to tumor heterogeneity, which can affect initial treatment strategies, 60% to 75% of patients demonstrate local recurrence following tumor debulking and adjuvant therapy [6,7]. Recurrence in patients <6 months post initial therapy is said to be due to platinum-resistant tumors that are associated with poor long-term prognosis [8]. Ovarian tumor therapies have been well reviewed for the use of platinum taxanes as front-line therapeutics and for the potential of intraperitoneal chemotherapy to treat residual disease during surgery, as well as newer second-line therapies that target unique molecular mechanisms, including Trabectedin, poly-ADP ribose polymerase (PARP) inhibitors, and Bevacizumab, in patients showing reduced platinum sensitivity [5,6,7,9,10,11,12,13,14,15].

Targeted drug delivery systems can localize cytotoxic agents at tumor sites and could enhance current protocols by (i) overcoming non-specific toxicity, (ii) delivering a more effective dose per treatment cycle, and (iii) overcoming canonical multidrug resistance (MDR) pathways seen in incidences of recurrence. In many cases, compartmentalizing drugs in polymeric and organic nanoparticles (NPs) has shown potential for drug delivery. Drug-loaded nano-carriers have been developed using poly-lactic-co-glycolic acid (PLGA) [16], gold [17], silicon [18], and silver [19] to deliver drugs to tumor cells or to isolate and identify malignant cells in vitro and in vivo by fluorescent imaging [20,21]. The structure of NPs promotes long-term circulation throughout the body by reducing renal clearance. NPs can be used for active targeting but also readily accumulate due to the enhanced permeability and retention (EPR) effect because of inconsistencies in vascular pore size associated with tumors [22,23].

The amphipathic structure of NPs provides an ideal platform to load small molecules, and the large surface area can be used to functionalize the outer shell with a target-specific molecule, such as peptides, antibodies, and aptamers [24,25]. Aptamers are short single-stranded DNA or RNA oligonucleotides, 50–100 bases in length, that recognize and selectively bind to cell- and tissue-specific biomarkers with dissociation constants (Kd) measured in µM to pM ranges [26,27]. Aptamers (i) display a conventional binding behavior similar to that of antibodies but display little to no immunogenic effect, (ii) can be produced without the use of an animal model, (iii) allow for post transcriptional modifications that can alter targeting kinetics and prevent nuclease degradation, and (iv) remain stable for long periods even under adverse conditions [28,29,30,31,32,33].

Despite significant interest in aptamers in oncology and reports of their use both in vitro and in mouse models in vivo, no clinical trials to date have used aptamer-labeled nanoparticles to directly target cancer cells [34,35,36,37,38,39,40,41,42]. Nanoparticles loaded with chemotherapeutics are common in clinical trials and recognized to promote stability and reduce generalized toxicity; as of 2025, 180 clinical trials have investigated the role of administering drug-loaded NPs to patients with various disorders and as visual diagnostic aids (clinicaltrials.gov). Almost 50% (83 current and concluded trials) focus on patients with solid tumors and recurring malignancies. As of 2025, the majority of these ongoing cancer related trials utilize paclitaxel-loaded micelles (nab-paclitaxel, Abraxane ^®^, ABI-007) composed of a negatively charged albumin shell with a hydrophobic core that solubilizes lipophilic paclitaxel in saline (0.9% NaCl) [43]. In addition to cytotoxic drugs, NPs loaded with gene therapy plasmids have been successful in phase I and II trials, including an anti-cyclin G1 gene construct (Rexin-G) [44] and FUS1 tumor suppressor gene [45] in a stable lipid particle suspension. Additionally, the incorporation of siRNA into lipid-based NPs include phase I clinical trials that treat multiple myelomas, non-Hodgkin lymphoma, and non-specific liver tumors [46,47,48]. However, these remain reliant on the passive enhanced permeability and retention (EPR) effect to enter target cells [49,50].

Distinct expression surface receptors or biomarkers give individual cancer subtypes unique molecular signatures that may be identified and bound by aptamers [51,52]. When aptamers are covalently linked directly to drugs or drug carriers, the resulting aptamer–drug conjugate creates a circulating and actively targeting cytotoxic “smart bomb” [36]. As of 2025, of 730 trials with nanoparticles, only 4 labeled the nanoparticles—2 with amino acid peptides and 2 with protein analogs (somatostatin or folic acid); all 4 are active in Phase I, without reported results (https://clinicaltrials.gov/). As of 2025, of 63 clinical trials using aptamers, all are administered as the agent alone or directly conjugated to a chemotherapeutic (https://clinicaltrials.gov/). Thus, the approach to use aptamer-labeled nanoparticles to deliver chemotherapy is different from other approaches in oncology and remains untapped in the clinical setting.

Here, we describe a directed drug-loaded PLGA-based polymeric nano-carrier using a previously described EOC cell line-specific aptamer RLA01 identified by SELEX [53]. Labeling the surface of PLGA-based nanoparticles enhances target cell internalization while decreasing non-specific cell targeting both in vitro and in vivo. Further, RLA01 labeling of paclitaxel (Ptx)-loaded NPs significantly increases the induction of cell death by apoptosis. This technology has the potential to overcome the challenges of conventional systemic anti-cancer treatment regimens and lead to higher local doses, more effective tumor cell death, reduction in subsequent treatment rounds, and reduced relapse [54].

## 2. Materials and Methods

### 2.1. Cell Lines

The human ovarian adenocarcinoma cell lines Caov-3 (HTB-75), SK-OV-3 (HTB-77), and SW626 (HTB-78) (ATCC; Manassas, VA, USA) were maintained 37 °C 5% CO_2_. Caov-3 cell lines were maintained in Dulbecco’s minimal essential medium (DMEM, GIBCO, Waltham, MA, USA) supplemented with 10% fetal bovine serum (FBS, GIBCO, Waltham, MA, USA) and 1% penicillin−streptomycin (GEMINI, Sacramento, CA, USA). SK-OV-3 cell lines were maintained in McCoys5a media (ATCC, Manassas, VA, USA) supplemented with 10% FBS (heat inactivated, GIBCO, Waltham, MA, USA) and 1% penicillin−streptomycin (GEMINI, Sacramento, CA, USA). SW626 cell lines were maintained in Leibovitz media (ATCC, Manassas, VA, USA) supplemented with 10% FBS (heat-inactivated, GIBCO, Waltham, MA, USA), 1% penicillin–streptomycin (GEMINI, Sacramento, CA, USA), and 1% sodium bicarbonate (7.5% *w*/*v*, Cellgro, Lincoln, NE, USA). The pancreatic carcinoma cell line Hs766T (ATCC, HTB-134) and Suit-2 , human cervical adenocarcinoma cell line HeLA (ATCC, CCL-2), breast adenocarcinoma cell lines MCF-7 (ATCC, HTB-22) and MDA-MB-231 (ATCC, CRM-HTB-26), and murine embryonic fibroblast NIH/3T3 (ATCC, CRL-1658) were all maintained in DMEM supplemented with 10% FBS (heat-inactivated, GIBCO, Waltham, MA, USA) and 1% penicillin−streptomycin (GEMINI, Sacramento, CA, USA). Normal epithelial cell lines HEK-293 (ATCC, CRL-1573) were maintained in DMEM supplemented with 10% FBS (heat-inactivated, GIBCO, Waltham, MA, USA) and 1% penicillin−streptomycin (GEMINI, Sacramento, CA, USA). HPV immortalized human ovarian epithelial (HOSE 6-3) cells were maintained in Medium199/MCDB105 media (1:1, Sigma Aldrich, St. Louis, MO, USA) supplemented with 10% FBS (heat-inactivated, GIBCO, Waltham, MA, USA), 1% penicillin– streptomycin (GEMINI, Sacramento, CA, USA), and 1% sodium bicarbonate (7.5% *w*/*v*, Cellgro, Lincoln, NE, USA). All cell lines were obtained and initially expanded, frozen stocks were generated at passage 2–4, and experiments on expanded cell lines were performed at passage 6–12.

### 2.2. Fluorescein Diacetate-Loaded Nanoparticles

PLGA-FDA NPs were labeled with the aptamer RLA01 (PGLA-FDA-RLA01). FDA fluorescent signal is dependent upon hydrolysis of FDA by an intracellular esterase or acidic conditions, which allows the acetoxymethyl-ester to yield fluorescein. Degradation of the NP and hydrolysis of FDA is necessary for generation of fluorescent signal quantifiable by flow cytometry.

### 2.3. ICG, Paclitaxel, and Paclitaxel-Loaded Nanoparticles

ICG is a near-infrared (NIR) fluorescent dye that utilizes wavelengths in the range of 700−900 nm used in imaging, allowing for a high signal-to-background ratio, as well as providing a means to detect contrast material at a depth of several millimeters within tissue. Paclitaxel (Ark Pharm Inc., Chicago, IL, USA) was diluted in DMSO (Sigma Aldrich, St. Louis, MO, USA) at 50 mg/mL. Additional dilution of paclitaxel for working doses was performed in a 50:50 solution CremaphorEl and ethanol (Sigma Aldrich, St. Louis, MO, USA) at 1 mg/mL and diluted to working concentrations in PBS (1×). DMSO vehicle was used for negative controls in cell line experiments. CremaphorEl vehicle was used for negative controls in xenograft experiments.

### 2.4. Aptamer Labeling of PLGA Nanoparticles

RLA01 aptamer was previously identified by SELEX [53]. The sequence of RLA01 is as follows:

5′-CTCCTCTGACTGTAACCACGCGGAAAGCATCAGGGTTGAGCATAGGTAGTCCAGAAGCCA-3′. PLGA-PCL-PEG blank, fluorescein diacetate, or paclitaxel-loaded nanoparticle suspension (10 mg/mL) in H_2_O was mixed with 100 µL NuLink-BE2 (2 mg/mL) for 5 min at room temperature. 5′-Amino-6C was added to the 5′ end of the aptamer. The primary amine group on the C6 linker acted as a versatile handle for conjugation to the NP and reduced steric hindrance to allow for the attachment to nanoparticles. Addition of NuLink-BE2 to functionalize the terminal amine groups of protruding hydrophilic PEG molecules is necessary for aptamer attachment. Nanoparticles were centrifuged (21,000 rcf, 4 °C), washed (2×, H_2_O) and resuspended in 1 mL H_2_O. To the cleaned nanoparticles, 0.2% (by weight of NPs) 5′-amino-6C-RLA01 conjugated aptamer was added and was incubated overnight under constant agitation at 4 °C. Aptamer-labeled NPs were spun, washed with H_2_O (5×s), and resuspended at a working concentration (1 mg/mL).

### 2.5. Nanoparticle Aptamer Characterization

Attachment of aptamer RLA01 to the NP surface was shown by agarose (4%) gel electrophoresis in 1× TAE buffer. The DNA marker (50BP Marker, New England Biolabs, Ipswich, MA, USA) and free aptamer served as standards for a 60-base-pair band. A 50 μL aliquot of NPs (1 mg/mL) was centrifuged at 21,000 rcf and at 4 °C for 10 min. The supernatant was removed, and NPs were resuspended in 20 μL H_2_O. Samples were run at 150 V for 2 h. Gels were stained with ethidium bromide.

### 2.6. SEM Imaging

Lyophilized NPs were sampled, placed on silicon wafers (LVEM5, DeLong America, Montreal, QC, Canada), and placed in a glass vacuum. After complete evacuation of atmospheric gases, the chamber was flooded with Argon gas and excited with 10 mA voltage to promote gold sputtering of NPs. Imaging was performed on a JEOL SEM 6460LV (JEOL USA, Peabody, MA, USA) 10 kV.

### 2.7. Confocal and Light Microscopy

Coav-3 cell lines were plated on a 35, 0/10 mm glass bottom culture dish (Greiner Bioone, Monroe, NC, USA) (seeded at 5.0 × 10^4^ per well/plate 48 h prior, 37 °C, 5% CO_2_). A total of 5 µL of labeled or unlabeled fluorescein diacetate-loaded NPs (100–900 ng range) was added to 1 mL cell-specific media and incubated on target cells at 37 °C and 5% CO_2_ for 2 h and agitated slightly every 30 min. Endosomal internalization was observed at 30, 60, 90, and 120 min post initial treatment. At 30 min prior to the desired timepoints, cells were treated with the endosomal-specific stain pHrodo^®^ Red Transferrin Conjugate (Invitrogen, Waltham, MA, USA) according to the manufacturers’ protocol (25 μg/μL). Cells were washed with PBS (3×) and fixed with 2 mL heptane (1:8.25 PBS: 37% Formaldehyde (Sigma Aldrich, St. Louis, MO, USA) at 37 °C for 10 min. Subsequent staining with DAPI (10 ng/μL, 10 min) was performed following standard procedures. Imaging of the cells was performed with an Olympus FluoView 1000 (Olymous USA, Center Valley, PA, USA) confocal microscope using DAPI (blue), pHrodo^®^ Red (orange), and Cy5 (red) filters. For light microscopy, Caov-3 cells were seeded 5.0 × 10^6^ per well/plate on a 6-well plate (BD Falcon, Franklin Lakes, NJ, USA) and incubated at 37 °C and 5% CO_2_ for 48 h. A total of 25 µL of labeled or unlabeled fluorescein diacetate-loaded NPs (100–900 ng range) was added to 2 mL cell-specific media and incubated on target cells at 37 °C and 5% CO_2_ for 2 h and was agitated slightly every 30 min. Cells were imaged at 30, 60, 90, and 120 min timepoints and continued to incubate. Light microscopy was performed on a Zeiss AxioCamMRc (Oberkochen, Germany) microscope using an X-cite-series 120Q laser (Excelitas, Pittsburgh, PA, USA).

### 2.8. Flow Cytometry

Caov-3 cells were plated on 6-well plates (seeded at 1.0 × 10^6^ 48 h prior, 37 °C 5% CO_2_). A total of 25 µL of labeled or unlabeled fluorescein diacetate-loaded NPs (100–900 ng range) was added to 2 mL cell-specific media and incubated on target cells at 37 °C and 5% CO_2_ for 2 and up to 4 h and was agitated slightly every 30 min. Cells were then washed with PBS (2×), scraped in 1 mL 1× PBS, and filtered through a 35 μm nylon mesh cell strainer polystyrene tube (BD Falcon). Cells were subjected to flow cytometric analysis within 1 min, and fluorescent events were determined with a Becton Dickinson LSRFortessa Flow Cytometer (Becton Dickinson, Franklin Lakes, NJ, USA) by counting 50,000 events of the single-cell population. Aptamer alone and non-aptamer-labeled blank NPs were used as a negative controls with both Caov-3 and HOSE 6-3 cells. Gating of the background fluorescence was set using the negative controls to 0.01% of the single-cell (non-aggregated) population analyzed.

### 2.9. Cell Proliferation and Apoptosis Assays

A Vybrant MTT Cell Proliferation Assay Kit (Invitrogen, Eugene, OR, USA) was used according to the manufacturers’ protocol. Cells were seeded into 96-well plates (seeded at 5000 cells/well 48 h prior, 37 °C 5% CO_2_). A total of 50 µL of the indicated treatment (10 µM to 0.001 µM) was added to 150 µL cell-specific media. Cells where then washed in PBS (3×) and given fresh media. Cell proliferation was observed at 0, 4, 8, 24, and 48 h post initial treatment. Cell proliferation activity was evaluated by adding 10 μL MTT stock solution and incubating for 4 h at 37 °C and 5% CO_2_; then, 100 μL SDS-HCl solution was added and allowed to stand for 4–12 h at 37 °C and 5% CO_2_. The resulting solution was pipetted vigorously, and optical density (OD) was measured at 570 nm using a Multiskan GO Microplate Reader (Thermo Scientific, Waltham, MA, USA). Percentage viable cells at all timepoints was calculated per the manufacturer’s instructions from average OD readings of control wells versus treated wells (*n* = 3) at the indicated concentrations (Supplemental Table S1). Standard deviations are too small to be visible but are shown in Supplemental Table S1. Significance between treatment groups was calculated by two-way ANOVA (* indicates *p*-values *p* < 0.001).

Cells were treated with 0.1 µM concentrations of paclitaxel-loaded NPs for 4 h at 37 °C and 5% CO_2_. Cells were washed with PBS (3×) and incubated at 37 °C and 5% CO_2_. The cells were harvested after 0, 4, 8, 12, 24, and 48 h post initial treatment. Cell pellets were frozen and stored at −80 °C. Protein extraction from cell lysates was collected by a Total Protein Extraction Kit (CHEMICON International, Temcula, CA, USA). Protein concentrations were analyzed by Bio-Rad D_c_ Protein Assay (Bio-Rad Laboratories, Hercules, CA, USA). Western blotting: Equal amount (7 mg) of protein was subjected to electrophoresis on NuPAGE 10% Bis-Tris Gel (Life Technologies, Carlsbad, CA, USA) and then transferred to a positively charged nylon transfer membrane (GE Healthcare, Chicago, IL, USA). The blotted membranes were immune-stained with primary antibodies specific to Caspase-3 antigens (8G10, Cell Signaling Technology) or PARP1 antigens (H-300, Santa Cruz Biotechnology, Dallas, TX, USA) and then 2 secondary antibody (rabbit-IgG, Santa Cruz Biotechnology, Dallas, TX, USA). PARP-1 and caspase 3-treated membranes were stripped and immunoblotted with anti-β-actin. The signals were developed by an Amersham™ ECL Plus Western blotting Detection System (GE Healthcare, Chicago, IL, USA) according to the manufacturer’s protocol.

### 2.10. In Vivo Xenograft Studies

Nu/J female mice aged 6 weeks, ∼23 g (Jackson Laboratory; Bar Harbor, ME, USA), were inoculated with 5.0 × 10^6^ Caov-3 cells subcutaneously in the right-rear flank. Eight days post inoculation, mice were randomized into cohorts and began the predetermined treatment regimen. Mice either received weekly treatments via subcutaneous injection near the site of the tumor or received an injection via tail vein. Mice received weekly injections of either naked (*n* = 3) or RLA01 aptamer-labeled (*n* = 3) or ICG-loaded NPs (71 mg/kg nanoparticles, 0.5% ICG/0.36 mg/kg ICG) and were imaged at 1, 4, 8, 12 24, 48, 72, and 96 h post treatment. Negative control mice received injections of vehicle only (*n* = 3). All mouse imaging was performed on an IVIS^®^ Spectrum Pre-clinical In Vivo Imaging System (Perkin Elmer, Waltham, MA, USA) with appropriate wavelength (745 nm, excitation wavelength and filter; 810 nm, emission wavelength and filter). All animal experiments were performed in accordance with protocols evaluated and approved by the IBC committee at UNC Charlotte. Body weight was measured twice weekly. Tumor size was measured before administering weekly treatment by digital calipers. Body weights and tumor size measurements were blinded, i.e., obtained by personnel without knowledge of the treatment group. As per IBC guidelines, mice were monitored for complications daily that would warrant humane endpoints to the protocol. Sixty days following initial inoculation, mice were sacrificed according to IBC guidelines.

## 3. Results

### 3.1. Labeling the Surface of Nanoparticles with Aptamer RLA01

Aptamer RLA01 was previously identified by SELEX using Caov-3 cells against HOSE 6-3 cells [53]. UNAfold was used to predict energetically stable tertiary structures of RLA01, and top energetically stable structures were determined by the Gibbs free energy equation (Supplemental Figure S1). Specificity of binding and internalization of RLA01 were further demonstrated with Caov-3 and SK-OV-3 but not with a large panel of immortalized nonmalignant epithelial and multiple malignant epithelial cell lines, including malignant pancreatic epithelial carcinomas (Suit-2, Hs766t), mammary epithelial adenocarcinomas (MCF-7, MDA-MB-231), cervical epithelial adenocarcinoma (HeLa), and kidney epithelial cells (HEK293) [53].

**Figure 1:**
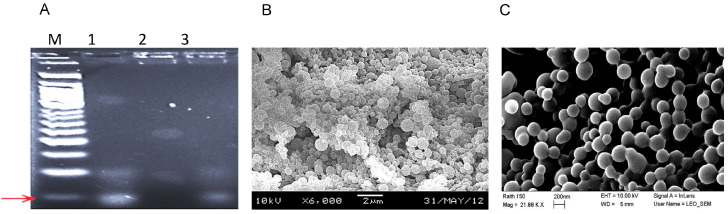
Aptamer RLA01 labeling of PLGA nanoparticles. (**A**) PAGE electrophoresis demonstrating the attachment of RLA01 to nanoparticles and SEM imaging showing nanoparticle size. The labeling of NPs with Caov-3-specific aptamer RLA01 is shown by agarose electrophoresis. Lane (M), 50 bp weight marker; lane (1), RLA01 (60 bp) stock and PLGA-NP with NuLink-BE2; lane (2), NP and RLA01 aptamer mixed in absence of NuLink-BE2; lane (3), PLGA-NP-RLA01 post-boiling. Red arrow indicates RLA01 NP conjugate. (**B**) SEM imaging of loaded NPs at indicated magnification. (**C**) Dynamic light scattering analysis of spherical NPs within the range of 200–250 nm. Original figures can be found in supplementary materials.

5′-Amino-6C was added to the 5′ end of the aptamer. The primary amine group on the C6 linker acted as a versatile handle for conjugation to the NP and reduced steric hindrance to allow for the attachment to nanoparticles. PGLA-PEG NP surfaces were labeled with aptamer RLA01 (PLGA-Apt) with NuLink-BE2 (Figure 1A, lane 1) and showed an electrophoresis band at a molecular weight of 60 bp, as expected. NPs incubated with RLA01 in the absence of NuLink-BE2 failed to label the surface of NPs, demonstrating the necessity of NuLink-BE2 to attach RLA01 to the NP surface (Figure 1A, lane 2). RLA01-labeled NPs denatured by boiling showed a band (Figure 1A, lane 3). These data confirm the labeling of the NP surface with aptamer RLA01. Scanning electron microscopy (SEM) (Figure 1B) and dynamic light scattering analysis (Figure 1C) showed NPs to be within the range of 200–250 nm and showed spherical NPs, in solution and lyophilized, respectively, with a high degree of size uniformity.

### 3.2. Cellular Uptake of Aptamer-Labeled Nanoparticles

**Figure 2:**
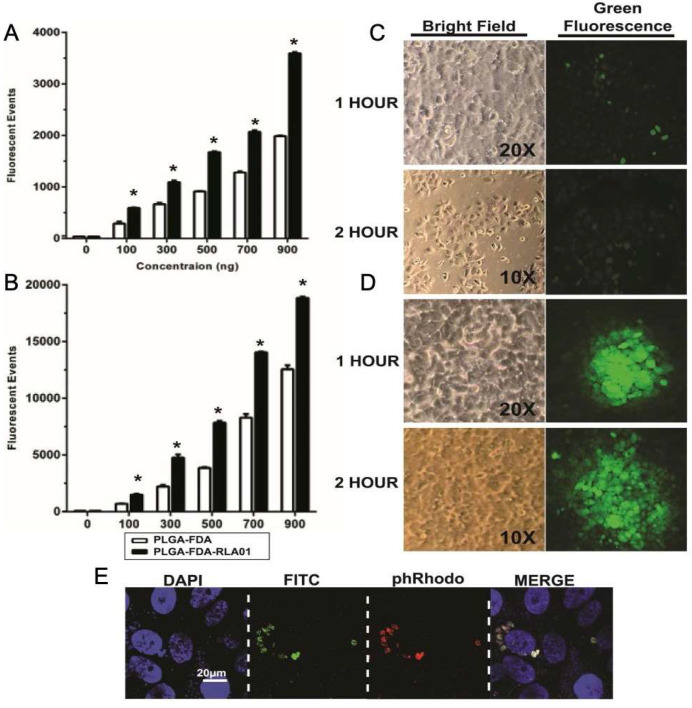
Cellular internalization of RLA0-labeled FDA-loaded PLGA nanoparticles in Caov-3 cells. (**A**) Fluorescent events quantified by flow cytometry in Caov-3 cell populations with increasing concentrations of 0–900 ng at 1 h. Data points represent the average total fluorescence (n = 3, error bars ± SD). White bars: FDA-loaded naked PLGA nanoparticles. Black bars: FDA-loaded PLGA nanoparticles labeled with RLA01. * *p*-value < 0.001. (**B**) Fluorescent events quantified by flow cytometry in Caov-3 cell populations with increasing concentrations of 0–900 ng at 4 h. Data points represent the average total fluorescence (n = 3, error bars ± SD). White bars: FDA-loaded naked PLGA nanoparticles. Black bars: FDA-loaded PLGA nanoparticles labeled with RLA01. * *p*-value < 0.001, (**C**) Qualitative analysis by inverted microscopy of naked FDA-loaded NPs at 1 h and 2 h of incubation with Caov-3 cells. (**D**) Qualitative analysis by inverted microscopy of RLA01-labeled FDA-loaded NPs at 1 h and 2 h of incubation with Caov-3 cells. (**E**) Confocal immunofluorescent imaging of Caov-3 cells (60×) after incubation with RLA01-labeled FDA-loaded PLGA nanoparticles 1 h. Nuclear stain (DAPI-blue), fluorescein diacetate (FDA)-loaded NPs (green), endosomal-specific marker pHrodo^®^ Red Transferrin Conjugate (pseudo-colored red), and merge.

Fluorescein diacetate-loaded NPs (PLGA-FDA) were labeled with aptamer RLA01 (PGLA-FDA-RLA01) to assess internalization into target EOC Caov-3 cells. Identification of fluorescent signal is dependent upon hydrolysis of FDA by an intracellular esterase or acidic conditions, which allows the acetoxymethyl-ester to yield fluorescein. Degradation of the NP and hydrolysis of FDA is necessary for generation of fluorescent signal quantifiable by flow cytometry. Internalization of NPs was determined by observed fluorescent events via flow cytometry and fluorescent microscopy (Figure 2). We observed a dose-dependent increase in FDA-positive cells within the total cell population when Caov-3 cells were incubated with RLA01 aptamer-labeled versus naked NPs (Figure 2A,B). Although both aptamer-labeled and naked NPs internalize into cells in a time-dependent and dose-dependent manner, the fluorescent events observed with aptamer-labeled NPs were significantly increased by 20–85% depending on the dose (two-way ANOVA, significant *p*-values *p* < 0.001) than with non-labeled NPs for all 5 treatment doses in both 1 h and 4 h timepoints (Figure 2A,B). Immunofluorescent microscopy demonstrated the cellular uptake and sub-cellular localization of NPs. Although naked FDA-loaded nanoparticles showed some minimal fluorescent uptake at the 1 and 2 h timepoints (Figure 2C), significantly more fluorescent uptake was observed with RLA01-labeled FDA-loaded nanoparticles (Figure 2D). Confocal microscopy was used to characterize the mechanistic pathway of RLA01-labeled nanoparticle cellular internalization (Figure 2E). The endosomal internalization of FDA-loaded NPs was shown using the endosomal-specific marker pHrodo red transferrin conjugate, whose fluorescent intensity is dependent upon pH. The fluctuating acidity within the endosome caused by the rupturing NP would alter the acidity levels in the endosome and thus reduce or enhance the fluorescent intensity observed. Spherical endosomal structures co-localized with FDA green fluorescence indicative of cleaved FDA, suggesting endosomal internalization of RLA01-labeled NPs (Figure 2E). In addition, the loaded NPs accumulate into the cytoplasm of the cell proximal to the nucleus (DAPI) in Caov-3 cells (Figure 2E).

**Figure 3:**
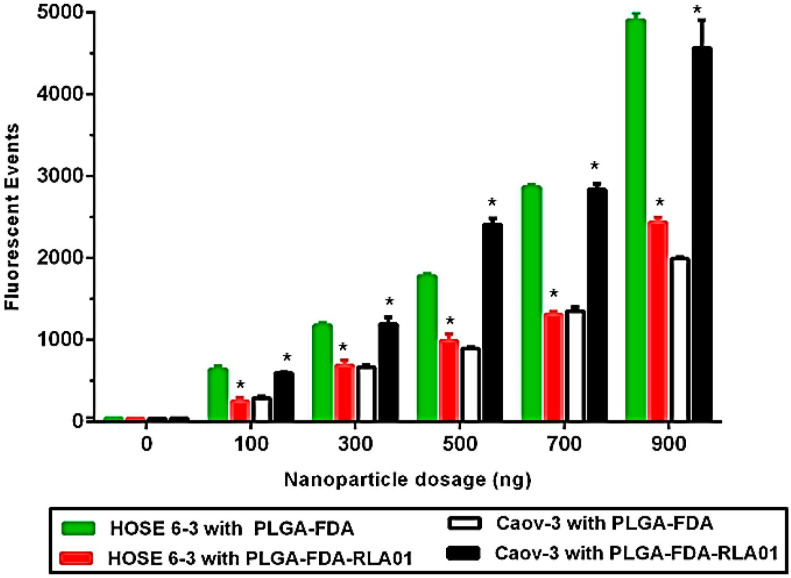
RLA01 labeling of nanoparticles inhibits random internalization in non-malignant immortalized epithelial cells. Caov-3 and non-malignant ovarian epithelial HOSE 6-3 cells were treated with increasing concentrations of 0–900 ng RLA01-labeled and naked FDA-loaded NPs for 2h. Data points represent the average total fluorescence (n = 3, error bars ± SD). Green bars: FDA-loaded naked PLGA nanoparticles with HOSE 6-3 cells. Red bars: RLA01-labeled FDA-loaded PLGA nanoparticles with HOSE 6-3 cells. White bars: FDA-loaded naked PLGA nanoparticles with Coav-3 cells. Black bars: RLA01-labeled FDA-loaded PLGA nanoparticles with Caov-3 cells. * *p*-value < 0.001.

Interestingly, RLA01 labeling of nanoparticles inhibited random internalization in non-malignant immortalized epithelial cells. Caov-3 and non-malignant ovarian epithelial HOSE 6-3 cells were treated with increasing concentrations of 0–900 ng RLA01-labeled and naked FDA-loaded NPs for 2 h. When Caov-3 cells were incubated with RLA01 aptamer-labeled versus naked NPs, we observed a significant increase in FDA-positive cells at all doses tested (Figure 3, *p*-value < 0.001). By contrast, when HOSE 6-3 cells were incubated with RLA01 aptamer-labeled versus naked NPs, we observed a significant decrease in FDA-positive cells at all doses tested (Figure 3, *p*-value < 0.001). These data suggest that the presence of the EOC-specific aptamer on the nanoparticle surface reduces aptamer–NP interactions with the cell membrane and may enhance internalization into target cancer cell populations in a heterogeneous population of cells analogous to the tumor microenvironment in vivo.

### 3.3. Targeted Efficacy of Cell Killing with RLA01-Labeled Paclitaxel-Loaded Nanoparticles

**Figure 4:**
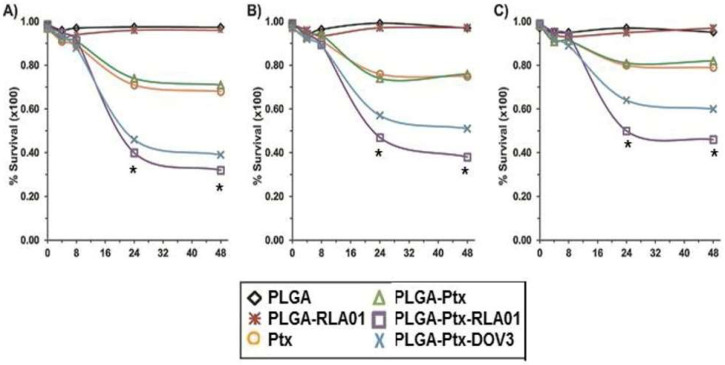
RLA01 labeling of nanoparticles induces cell killing by paclitaxel-loaded nanoparticle treatments of Caov-3 cells. Graphs represent a complete 48 h timeline of cell proliferation after treatment with NPs using an MTT proliferation kit to assess cell viability post treatment at 0, 4, 8, 12, 24, and 48 h: (**A**) 0.1 µM; (**B**) 0.01 µM; (**C**) 0.001 µM concentrations by weight of paclitaxel. Black diamonds: naked PLGA nanoparticles; yellow circles: paclitaxel alone; red stars: RLA01-labeled empty PLGA nanoparticles; green diamonds: naked PLGA nanoparticles loaded with paclitaxel; purple squares: RLA01-labeled nanoparticles loaded with paclitaxel; blue: X-DOV3-labeled nanoparticles loaded with paclitaxel. Data points represent the calculated percent cell survival per manufacturer’s instructions from average OD reading recorded (n = 3). * Two-way ANOVA, *p*-values < 0.001. Error bars too small to be visible in figure; standard deviations are provided in Supplemental Table S1.

We sought to determine whether RLA01 aptamer-labeled NPs loaded with the platinum taxane paclitaxel (Ptx) could increase cellular uptake and thus increase the efficacy of Ptx-induced cell death (Figure 4). Caov-3 cell lines were exposed to increasing molar concentrations (by weight of Ptx) of Ptx alone or in different combinations/formulations of RLA01-labeled NPs. Cells were treated for 4 h then analyzed by MTT cell proliferation assay at 0, 4, 8, 24, and 48 h post-treatment. Cohorts included unloaded naked NPs (PLGA), unloaded NPs labeled with RLA01 (PLGA-Apt), Ptx alone (Ptx), Ptx-loaded unlabeled NPs (PLGA-Ptx), and Ptx-loaded NPs labeled with RLA01 (PLGA-Ptx-Apt). Naked unloaded NPs enabled determination of proliferation effects caused by the PLGA-based polymeric vehicle. Unloaded NPs labeled with RLA01 allowed determination of whether the increased uptake of PLGA NPs can lead to adverse effects on Caov-3 cell lines independent of Ptx. Additionally, a previously described aptamer [55] was conjugated to Ptx-loaded NPs (PLGA-Ptx-AptDOV3) in order to compare models with an established aptamer identified by similar methods. PLGA alone and PLGA-Apt had no apparent effect on cell proliferation (Figure 4A–C). PLGA-Ptx treatment was as effective as Ptx alone when treated with equal doses, showing ~25% reduction in cell viability when compared to PLGA treatments. RLA01-labeled PLGA-Ptx treatments caused a significant decrease in cell proliferation when compared to either PLGA-Ptx or Ptx alone at 24 and 48 h timepoints (Figure 4A–C). RLA01-labeled NPs loaded with Ptx caused a significant > 50% reduction in cell proliferation for all three treatment concentrations (calculated by two-way ANOVA, *p*-values *p* < 0.001).

Caspase-3 and PARP1 protein levels confirmed that the observed anti-proliferation effect observed by PLGA-Ptx-Apt treatment was due to apoptosis signaling [56,57]. Negative control cells showed low to undetectable levels of caspase-3 and PARP1 while a time-dependent increase in both PARP1 and Caspase-3 occured following treatment with RLA01-labeled PLGA-Ptx nanoparticles consistent with MTT proliferation data.

### 3.4. Xenograft Model to Assess In Vivo Accumulation and Honing of RLA01-Labeled Nanoparticles to EOC Tumors

A xenograft mouse model was generated by injection of 5 × 10^6^ Caov-3 cells in the rear flank of female Nu/J mice (n = 9). Mice either received weekly treatments via subcutaneous injection near the site of the tumor or received injection via tail vein to determine the potential for RLA01 to direct nanoparticles through the blood stream to the site of tumor.

**Figure 5:**
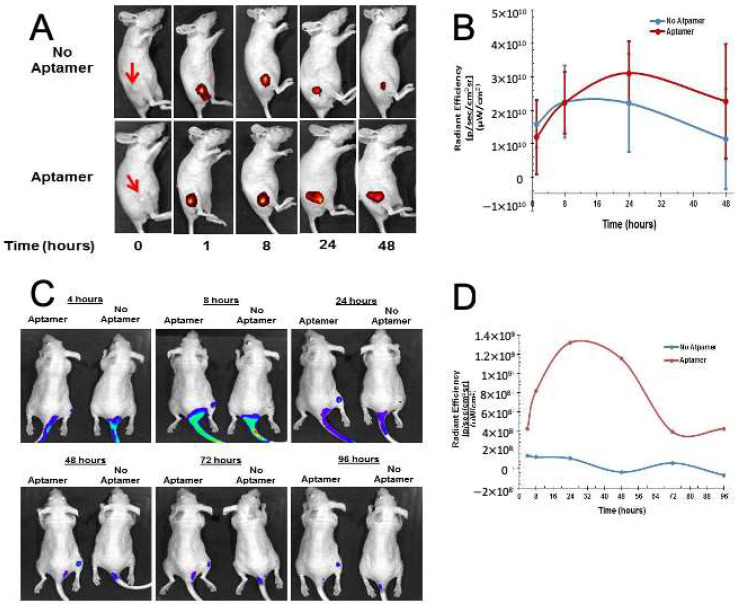
Retention of RLA01-labeled versus naked ICG-loaded nanoparticles in vivo. Female Nu/J mice were inoculated with 5.0 × 10^6^ Caov-3 cells in the right rear flank (red arrow). (**A**) After tumor burden reached 0.67 mm, mice were injected near the site of the tumor with either RLA01 aptamer-labeled (top) or naked ICG-loaded NPs (bottom). (**B**) Graphical representation of radiant efficiency over time measured by IVIS showing increased signal in mice injected with RLA01-labeled ICG-loaded nanoparticles (*n* = 3) versus naked ICG-loaded nanoparticles (*n* = 3) at 24 and 48 h timepoints post injection. (**C**) After tumor burden reached 0.67 mm, mice were injected via tail vein with either RLA01 aptamer-labeled or naked ICG-loaded NPs. ICG signal was observed in mice injected with RLA01-labeled nanoparticles over 96 h. (**D**) Graphical representation of radiant efficiency at the site of the xenograft tumor over time with data points directly taken from the images and analysis of 3C. Signals measured by IVIS showing a trend of increased signal in mouse injected with RLA01-labeled ICG-loaded nanoparticles versus naked ICG-loaded nanoparticles at 24, 48, 72, and 96 h timepoints post injection via tail vein.

Mice received weekly subcutaneous injections of either naked (n = 3) or RLA01 aptamer-labeled (n = 3), ICG-loaded NPs (71 mg/kg nanoparticles, 0.5% ICG/0.36 mg/kg ICG) and were imaged at 1, 4, 8, 12, 24, 48, 72, and 96 h post treatment. Vehicle-treated (n = 3) mice were negative controls. IVIS imaging signals were used to compare the radiant efficiency observed after treatment with aptamer-labeled ICG-loaded NPs (PLGA-ICG-RLA01) versus non-aptamer-labeled ICG-loaded NPs (PLGA-ICG). IVIS imaging and graphical representation of radiant efficiency over time showed increased signal in mice injected with RLA01-labeled ICG-loaded nanoparticles versus naked ICG-loaded nanoparticles at 24 and 48 h timepoints post injection (Figure 5A,B). These proof-of-principle data are consistent with efficient retention of RLA01-labeled NPs at the site of the tumor over time. However, a larger cohort of mice would be required to demonstrate a consistent and statistical difference between RLA1-labeled and naked NP retention.

IVIS imaging and graphical representation of radiant efficiency over time showed increased signal in mice injected via tail vein with RLA01-labeled ICG-loaded nanoparticles (n = 3) versus naked ICG-loaded nanoparticles (n = 3) at 24, 48, 72, and 96 h timepoints post injection (Figure 5C,D). Fluorescent ICG was used to monitor the circulatory kinetics of NPs and any localization and retention within the mouse. The graphical data shown highlights the radiant efficiency in a specific region of interest (ROI). The radiant efficiency and total area of detectable fluorescence diminished at 24 h with non-labeled nanoparticle treatments. The slow drainage and clearance of NPs away from the injection site is likely due to the lack of vascularization and slow absorption of NPs into the blood stream.

At 4 h post injection via tail vein, RLA01-labeled ICG NPs began to localize at the tumor site. At 8 h post injection, radiant efficiency rose by 25% in RLA01-labeled NPs, suggesting absorption at the injection site (Figure 5C). At the tumor site, mice treated with RLA01-labeled NPs displayed a higher fluorescent signal and larger area of fluorescence, suggesting accumulation at the tumor site (Figure 5C). At 24 and 48 h post injection, the mouse receiving RLA01-labeled NPs showed a persistent fluorescent signal at the tumor while the mouse receiving naked NPs displayed no detectable fluorescence level above background (Figure 5C,D). These proof-of-principle data are consistent with efficient honing of RLA01-labeled NPs through blood vessels to the site of tumor and retention of RLA01-labeled NPs at the site of the tumor over time. However, a larger cohort of mice would be required to demonstrate consistent and statistical difference between RLA1-labeled and naked NP honing.

## 4. Discussion

We previously described the binding and internalization kinetics of aptamer RLA01 alone when incubated with target Caov-3 cell lines [53]. RLA01 demonstrates a high specificity for targeting malignant Caov-3 cells and the ability to internalize by the endocytic pathway. Thus, RLA01 could promote internalization of NPs that have been labeled with RLA01 on its surface. Additionally, this would suggest that loading NPs with small molecules such as cytotoxic drugs could increase its internalization into target cells.

Immunofluorescent microscopy and flow cytometry data suggest that FDA-loaded RLA01 aptamer-labeled NPs show a significantly higher rate of internalization over time and produce cell populations with more robust fluorescent activity when compared to the non-aptamer-labeled NP treatment. Labeling the surface of NPs with EOC-specific RLA01 increased the cellular uptake of FDA-loaded NPs as compared to non-labeled NPs when incubated on Caov-3 cells. RLA01 promotes internalization by receptor–aptamer binding at the membrane following washing of residual particles, suggesting high-affinity bonds made between the aptamer and cell surface moieties.

Although nonspecific NP internalization occurs through EPR effects, we show that RLA01-labeled NPs occurs through active receptor-mediated endocytosis. Interestingly, the data show that RLA01 labeling of NPs results in reduced uptake in non-malignant cells (HOSE 6-3). These data suggest that the presence of the EOC-specific aptamer RLA01 on the nanoparticle surface creates an inhibitory barrier to passive interactions with the cell membrane of non-target cells, and this inhibition may further promote the specific binding and internalization into target cancer cell populations. This provides an additional specificity and added potential for targeted therapy approaches in a heterogeneous microenvironment in vivo.

These data demonstrate increased cell death at equitoxic dosages with RLA01 aptamer-labeled Ptx-loaded NPs versus Ptx alone, as well as non-labeled Ptx NPs, in cells. Use of aptamer-directed NPs caused a statistically significant enhanced incidence of Ptx-induced cell death at all doses examined. We show an increased incidence of internalization with aptamer-labeled NPs in vitro, correlated with in vivo assays. This evidence would suggest that aptamer binding and internalization are not significantly altered when comparing in vitro and in vivo models.

The PLGA polymer allows for the encapsulation of multiple hydrophobic drugs, such as Ptx, because of the amphipathic nature of the particle. In addition to being biodegradable and showing high biocompatibility, PLGA is found in a variety of FDA-approved therapeutic devices and products. The successful labeling of NPs with aptamers demonstrates the feasibility of using them as a labeling platform, and it could be altered in several ways, including the dual loading with both ICG and Ptx for in vivo studies. Since ICG is approved by the FDA, the clinical translation of ICG-based imaging is rapidly advancing and used in clinical trials to image colorectal, head and neck, and breast cancers. Dual targeting and combinatorial loading of NPs could potentially lead to increased sensitivity in diagnostics or treatments that circumvent multidrug resistance pathways. Furthermore, generating tumor-specific aptamers could help differentiate between various tumor subtypes. Multi-targeted aptamers specific to tumor sub-types could increase the positive identification of malignant tissue with more accuracy than PCR identification of biomarkers in serum. This could increase the general utility of Cell-SELEX-identified aptamers by employing them to assess specific information about the cell surface proteome.

Despite all mice growing palpable tumors, there was significantly less max fluorescent signal after both 24 and 48 h in mice who received naked ICG-loaded NP treatments when compared to RLA01 aptamer-labeled NP treatments. These data suggest increased localization and retention at tumors, which can minimize cytotoxic effects to neighboring or distal non-malignant tissue with proper dosing and direct administration into malignant tissue. However, a larger cohort of mice would be required to demonstrate consistent and statistical difference between RLA1-labeled and naked NP honing and retention. As measured by daily weights, physical appearance, activity, and histologic analysis, mice showed no evidence of systemic toxicity upon treatment with NPs nor NPs labeled with aptamer RLA01, even at high dosages, as compared to those treated with vehicle only. RLA01 aptamer-labeled NPs administered by tail vein injection promoted the accumulation of NPs at the tumor. The imaging data clearly indicates that ICG-loaded NPs administered by tail vein are present within the mouse up to and beyond 48 h, suggesting no apparent increased degradation of NPs in an in vivo model. The increased retention of drugs or NPs at the tumor site is dependent on two important factors: (i) circulation time with regard to kidney filtration, meaning larger molecules will remain in circulation longer, and (ii) development of tumor masses large enough where vascularization is essential for sustained tumor growth.

## 5. Conclusions

Overall, we established that this RLA01-labeled NP model was designed efficiently and that labeled NPs are useful vectors for delivering targeted therapeutics in mouse models. These data demonstrate the effective combinatorial use of aptamer RLA01 and nanoparticle technologies for the direct targeting of tumor cell populations both in vitro and in vivo. As aptamer screening techniques continue to be optimized, they will provide the ability to identify aptamers unique to each individual’s tumor and development of personalized medicine.

## 6. Patents

Patent No. 11008575. Approved week of 18 May 2021. DNA aptamers against cancer and the uses thereof in delivery of therapy and diagnosis of cancer. Inventors: C. Richardson and G. Benedetto.

## Data Availability

The original contributions presented in this study are included in the article/Supplementary Materials. Further inquiries can be directed to the corresponding author.

## Supplementary Materials

The following supporting information can be downloaded at https://www.mdpi.com/article/10.3390/biom15081123/s1: Figure S1: Predicting energetically stable tertiary structures of RLA01. To predict probable secondary structures, nucleotide sequences were analyzed by UNAFold software (version 1). The top energetically stable structures as determined by the Gibbs free energy equation analyzed at 20 °C. Corresponding ΔG (kcal×mole^−1^) values for each structure are as follows: A −2.4; B −2.34; C −2.24; D −2.08; E −1.47; F −1.43.; Table S1: MTT raw values for OD readings and standard deviations (SDs).
