# Supplementary material for: Aptamer-Coated PLGA Nanoparticles Selectively Internalize into Epithelial Ovarian Cancer Cells In Vitro and In Vivo
Source: Biomolecules. 2025 Aug 4;15(8):1123. doi: 10.3390/biom15081123 (PMC12383946; doi:10.3390/biom15081123)
Supplement: Supplementary file 1 [file biomolecules-15-01123-s001.zip › biomolecules-3761562-supplementary.pdf]

Supplemental Table S1. MTT raw values for OD readings and standard deviations (SD)

Caov3 0HR

REP.	.1 µM	.01 µM	.001 µM	CONTROL WELLS				
	**BLANK NPs**							
1	0.6278	0.5983	0.5864	0.6258	0.6149	0.6156	0.5736	**0.60748**
2	0.5819	0.5901	0.6427	0.5718	0.6105	0.6471	0.5176	**0.58675**
3	0.5858	0.6171	0.5745					
**AVG.**	**0.5985**	**0.60183**	**0.6012**					
**SD**	0.02545	0.01384	0.03643					
**% LIVE**	0.98523	0.99071	0.98967					
	**TAXOL NPs**							
1	0.5948	0.5482	0.5891	0.6068	0.6185	0.6244	0.6118	**0.61538**
2	0.6187	0.6998	0.6101	0.6118	0.5698	0.6761	0.6256	**0.62083**
3	0.5714	0.6018	0.6203					
**AVG.**	**0.59497**	**0.6166**	**0.6065**					
**SD**	0.02365	0.07688	0.01591					
**% ALIVE**	0.96684	1.00199	0.98558					
	**TAXOL**							
1	0.598	0.5844	0.579	0.5499	0.4945	0.6154	0.5879	**0.56193**
2	0.5863	0.5426	0.5841	0.5614	0.5988	0.6211	0.615	**0.59908**
3	0.5399	0.6457	0.6128					
**AVG.**	**0.57473**	**0.5909**	**0.59197**					
**SD**	0.03073	0.05186	0.01822					
**% LIVE**	1.02279	0.98635	1.05346					
	**BLANK NP-Aptamer**							
1	1.6619	1.719	1.7931	1.6872	1.6817	1.7481	1.6234	**1.6851**
2	1.738	1.6092	1.6302	1.6121	1.6312	1.7461	1.6424	**1.65795**
3	1.6236	1.661	1.6785					
**AVG.**	**1.6745**	**1.66307**	**1.7006**					
**SD**	0.05823	0.05493	0.08367					
**% LIVE**	0.99371	0.98692	1.0092					
	**TAXOL NP-Aptamer**							
1	1.4325	1.5847	1.4396	1.5055	1.4843	1.5881	1.3851	**1.49075**
2	1.4203	1.5232	1.5271	1.4119	1.6679	1.4994	1.4269	**1.50153**
3	1.5318	1.437	1.4865					
**AVG.**	**1.46153**	**1.51497**	**1.4844**					
**SD**	0.06116	0.07419	0.04379					
**% ALIVE**	0.97337	1.00895	0.98859					

Caov3 4HR

REP.	.1 µM	.01 µM	.001 µM	CONTROL WELLS

Supplemental Table S1. MTT raw values for OD readings and standard deviations (SD)

	**BLANK NPs**							
1	0.6067	0.6148	0.6018	0.686	0.6845	0.6214	0.6705	**0.6656**
2	0.5984	0.6358	0.6197	0.701	0.6715	0.5998	0.6247	**0.64925**
3	0.6211	0.5849	0.6339					
**AVG.**	**0.60873**	**0.61183**	**0.61847**					
**SD**	0.01149	0.02558	0.01609					
**% LIVE**	0.93759	0.94237	0.95259					
	**TAXOL NPs**							
1	0.6713	0.7802	0.7805	0.8215	0.7159	0.9114	0.8459	**0.82368**
2	0.7905	0.7194	0.6701	0.9112	0.7201	0.8998	0.8115	**0.83565**
3	0.7175	0.6945	0.7903					
**AVG.**	**0.72643**	**0.73137**	**0.74697**					
**SD**	0.0601	0.04409	0.06675					
**% ALIVE**	0.88194	0.88793	0.90687					
	**TAXOL**							
1	0.5649	0.5919	0.5886	0.6119	0.6488	0.6197	0.6943	**0.64368**
2	0.5919	0.6578	0.5716	0.6178	0.6249	0.6894	0.6058	**0.63448**
3	0.5823	0.5546	0.6184					
**AVG.**	**0.5797**	**0.60143**	**0.59287**					
**SD**	0.01369	0.05226	0.02369					
**% ALIVE**	0.90061	0.93437	0.92107					
	**BLANK NP-Aptamer**							
1	0.8238	0.7676	0.7547					
2	0.7895	0.8628	0.6927	0.8575	0.8536	0.8487	0.7749	**0.83368**
3	0.7013	0.7728	0.8659	0.849	0.8324	0.871	0.7121	**0.81613**
**AVG.**	**0.77153**	**0.80107**	**0.7711**					
**SD**	0.0632	0.05353	0.08776					
**% LIVE**	0.92546	0.96089	0.92494					
	**TAXOL NP-Aptamer**							
1	0.7518	0.7676	0.7898					
2	0.6995	0.6518	0.7349	0.8419	0.8216	0.8487	0.7912	**0.82585**
3	0.7413	0.7828	0.6806	0.8549	0.8324	0.8871	0.7621	**0.83413**
**AVG.**	**0.73087**	**0.73407**	**0.7351**					
**SD**	0.02767	0.07165	0.0546					
**% ALIVE**	0.87621	0.88004	0.88128					

Caov3 8HR

**REP.**	**.1 µM**	**.01 µM**	**.001 µM**		**CONTROL WELLS**			
	**BLANK NPs**							
1	1.6902	1.6768	1.6124	1.6197	1.7427	1.7435	1.6389	**1.6862**

Supplemental Table S1. MTT raw values for OD readings and standard deviations (SD)

2	1.5498	1.5671	1.6637	1.6518	1.5597	1.7002	1.6812	**1.64823**
3	1.5789	1.5239	1.6018					
**AVG.**	**1.6063**	**1.58927**	**1.62597**					
**SD**	0.0741	0.07882	0.03311					
**% LIVE**	0.97456	0.96423	0.9865					
	**TAXOL NPs**							
1	1.8402	1.9477	1.9439	2.0183	2.2361	2.0937	1.8491	**2.0493**
2	1.8138	1.8549	1.888	1.7855	2.0187	2.1361	2.0886	**2.00723**
3	1.9132	1.9622	1.775					
**AVG.**	**1.85573**	**1.9216**	**1.86897**					
**SD**	0.05149	0.05822	0.08604					
**% ALIVE**	0.90554	0.93769	0.912					
	**TAXOL**							
1	1.3454	1.3724	1.3191	1.4124	1.3043	1.3756	1.2108	**1.32578**
2	1.3983	1.4655	1.4055	1.6517	1.6183	1.216	1.406	**1.473**
3	1.2976	1.3295	1.4119					
**AVG.**	**1.3471**	**1.38913**	**1.37883**					
**SD**	0.05037	0.06953	0.05183					
**% LIVE**	0.91453	0.94306	0.93607					
	**BLANK NP-Aptamer**							
1	0.9125	0.9916	0.9215	1.029	1.0284	1.0998	0.9981	**1.03883**
2	1.1205	0.9348	1.1081	1.2594	1.0024	1.187	1.211	**1.16495**
3	0.8949	0.9856	0.985					
**AVG.**	**0.97597**	**0.97067**	**1.00487**					
**SD**	0.12548	0.03121	0.09487					
**% LIVE**	0.93949	0.93439	0.96731					
	**TAXOL NP-Aptamer**							
1	0.9647	0.9046	0.9438	1.021	1.229	0.9911	1.124	**1.09128**
2	0.8841	0.9918	0.9811	1.2441	0.9967	1.233	1.3717	**1.21138**
3	0.8553	0.9115	0.9653					
**AVG.**	**0.90137**	**0.93597**	**0.9634**					
**SD**	0.05671	0.04848	0.01872					
**% ALIVE**	0.82598	0.85768	0.88282					

Caov3 24HR

**REP.**	**.1 µM**	**.01 µM**	**.001 µM**	**CONTROL WELLS**				
	**BLANK NPs**							
1	1.1298	1.2348	1.1184	1.1583	1.0025	1.4531	1.1123	**1.18155**
2	1.1486	1.2154	1.2548	1.2141	1.3563	1.4668	1.2147	**1.31298**
3	1.2141	1.0668	1.1263					

Supplemental Table S1. MTT raw values for OD readings and standard deviations (SD)

**AVG.**	**1.16417**	**1.17233**	**1.1665**					
**SD**	0.04425	0.09191	0.07657					
**% LIVE**	0.98529	0.9922	0.98726					
	**TAXOL NPs**							
1	0.9715	0.8691	0.9402	1.0269	1.0115	1.0256	1.1008	**1.0412**
2	0.7248	0.9334	0.8912	1.0259	1.1006	1.0948	1.147	**1.09208**
3	0.8844	0.8921	0.9629					
**AVG.**	**0.86023**	**0.8982**	**0.93143**					
**SD**	0.12511	0.03258	0.03665					
**% ALIVE**	0.78771	0.82247	0.8529					
	**TAXOL**							
1	0.6978	0.6841	0.8513	1.2908	1.0417	0.68	1.2124	**1.05623**
2	0.6356	0.7836	0.7917	1.0888	0.838	0.8752	0.8404	**0.9106**
3	0.7945	0.868	0.8328					
**AVG.**	**0.7093**	**0.77857**	**0.82527**					
**SD**	0.08007	0.09205	0.03051					
**% ALIVE**	0.67154	0.73712	0.78134					
	**BLANK NP-Aptamer**							
1	0.5241	0.5477	0.5669	0.5886	0.5573	0.5814	0.5967	**0.581**
2	0.5109	0.5748	0.4995	0.6183	0.5967	0.5517	0.5622	**0.58223**
3	0.6261	0.5703	0.5876					
**AVG.**	**0.5537**	**0.56427**	**0.55133**					
**SD**	0.06305	0.01452	0.04607					
**% LIVE**	0.95301	0.9712	0.94894					
	**TAXOL NP-Aptamer**							
1	0.4045	0.5298	0.4237	0.6518	0.6556	0.6449	0.6592	**0.65288**
2	0.4612	0.4676	0.5241	0.6541	0.6657	0.5887	0.5586	**0.61678**
3	0.3834	0.4171	0.4925					
**AVG.**	**0.41637**	**0.4715**	**0.4801**					
**SD**	0.04023	0.05645	0.05134					
**% ALIVE**	0.63774	0.72219	0.73536					

Caov3 48HR

**REP.**	**.1 µM**	**.01 µM**	**.001 µM**		**CONTROL WELLS**			
	**BLANK NPs**							
1	0.6713	0.7002	0.7105	0.7215	0.7159	0.7014	0.8459	**0.74618**
2	0.7905	0.7194	0.6901	0.7112	0.7201	0.6998	0.8115	**0.73565**
3	0.7175	0.6945	0.7303					
**AVG.**	**0.72643**	**0.7047**	**0.7103**					

Supplemental Table S1. MTT raw values for OD readings and standard deviations (SD)

**SD**	0.0601	0.01305	0.0201					
**% LIVE**	0.97354	0.94442	0.95192					
	**TAXOL NPs**							
1	0.4273	0.5297	0.5855	0.5961	0.5698	0.4542	0.5419	**0.5405**
2	0.4543	0.4266	0.5306	0.6281	0.5109	0.4367	0.5801	**0.53895**
3	0.4693	0.4652	0.4028					
**AVG.**	**0.4503**	**0.47383**	**0.5063**					
**SD**	0.02128	0.05209	0.09374					
**% ALIVE**	0.83312	0.87666	0.93673					
	**TAXOL**							
1	0.8659	0.9025	0.8765	0.942	0.9789	0.994	0.954	**0.96723**
2	0.7721	0.8946	0.9884	1.2971	1.0721	1.2179	1.0333	**1.1551**
3	0.8732	0.9534	0.9772					
**AVG.**	**0.83707**	**0.91683**	**0.94737**					
**SD**	0.05638	0.03191	0.06163					
**% ALIVE**	0.72467	0.79373	0.82016					
	**BLANK NP-Aptamer**							
1	0.552	0.5941	0.6193	0.6068	0.6185	0.5844	0.5908	**0.60013**
2	0.668	0.5592	0.5659	0.6281	0.6629	0.6432	0.5998	**0.6335**
3	0.5662	0.5777	0.5956					
**AVG.**	**0.5954**	**0.577**	**0.5936**					
**SD**	0.06327	0.01746	0.02676					
**% LIVE**	0.99213	0.96147	0.98913					
	**TAXOL NP-Aptamer**							
1	0.4945	0.4266	0.4817	0.6918	0.6556	0.6449	0.7092	**0.67538**
2	0.3862	0.4776	0.4711	0.6789	0.6761	0.6218	0.6618	**0.65965**
3	0.4534	0.5171	0.5125					
**AVG.**	**0.4447**	**0.47377**	**0.48843**					
**SD**	0.05467	0.04537	0.02151					
**% ALIVE**	0.65845	0.70149	0.7232					
	**TAXOL NP-Gregs Aptamer**							
1	0.3266	0.4055	0.4529	0.6918	0.6556	0.6449	0.7092	**0.67538**
2	0.3862	0.3846	0.4711	0.6789	0.6761	0.6218	0.6618	**0.65965**
3	0.3147	0.4138	0.4129					
**AVG.**	**0.3425**	**0.4013**	**0.44563**					
**SD**	0.03831	0.01505	0.02977					
**% ALIVE**	0.50713	0.59419	0.65983					

Supplemental Table S1. MTT raw values for OD readings and standard deviations (SD)

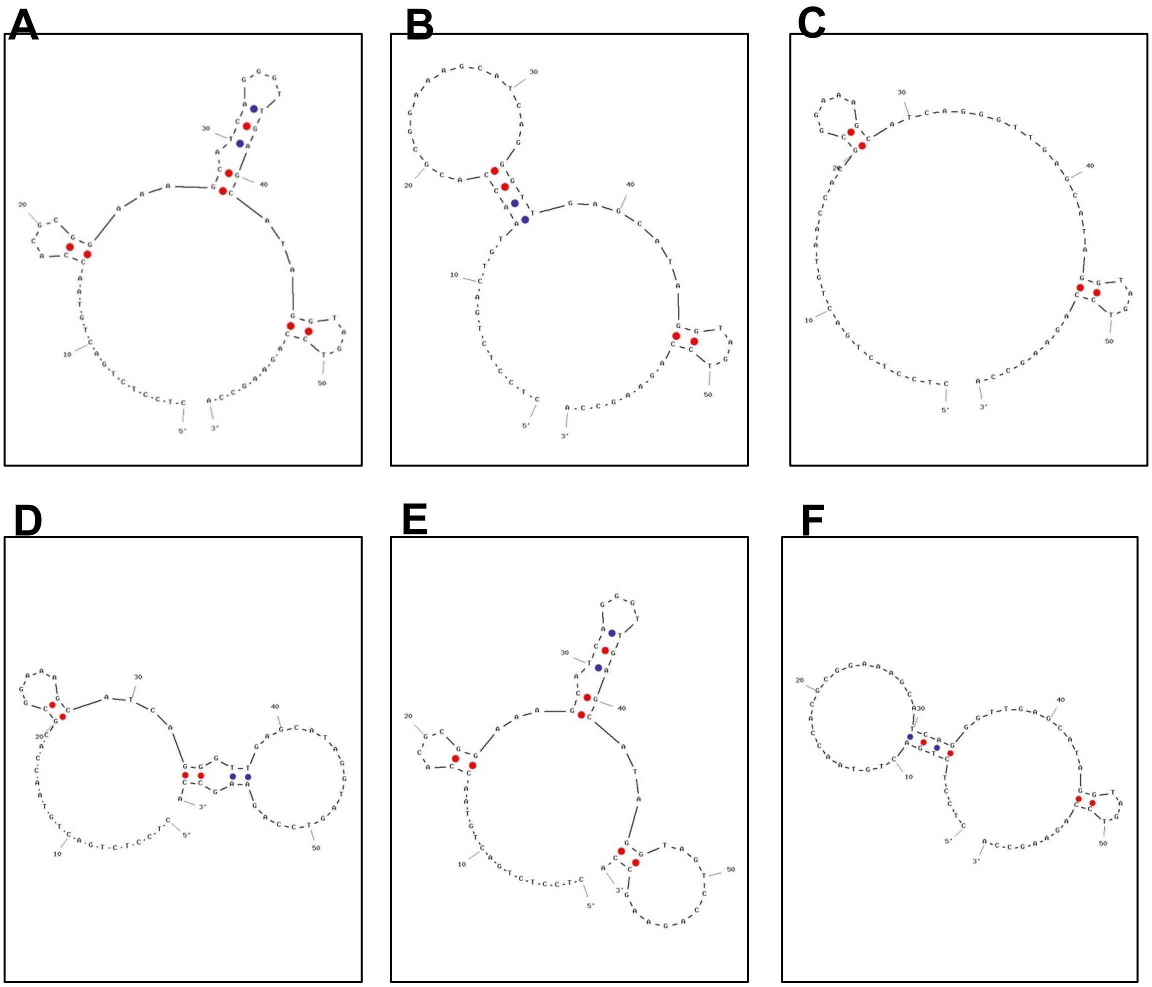

Supplemental Figure S1. Predicting energetically stable tertiary structures of RLA01. To predict probable secondary structures, nucleotide sequences. were analyzed by UNAFold software. The top energetically stable structures as determined by Gibbs free energy equation analyzed at 20°C. Corresponding ΔG (kcal*mole-1) values for each structure are: A -2.4; B -2.34; C -2.24; D -2.08; E -1.47; F -1.43.
