# Supplementary figures and images for: Aptamer-Coated PLGA Nanoparticles Selectively Internalize into Epithelial Ovarian Cancer Cells In Vitro and In Vivo
Source: Biomolecules. 2025 Aug 4;15(8):1123. doi: 10.3390/biom15081123 (PMC12383946; doi:10.3390/biom15081123)

Figure 1

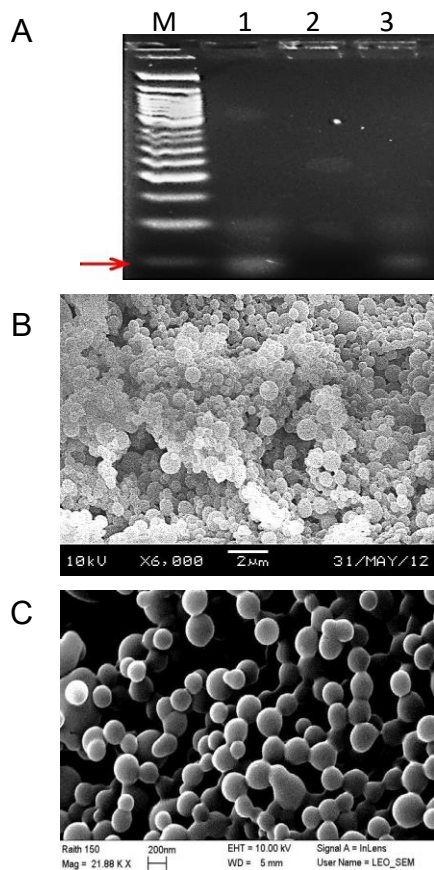

Figure 2

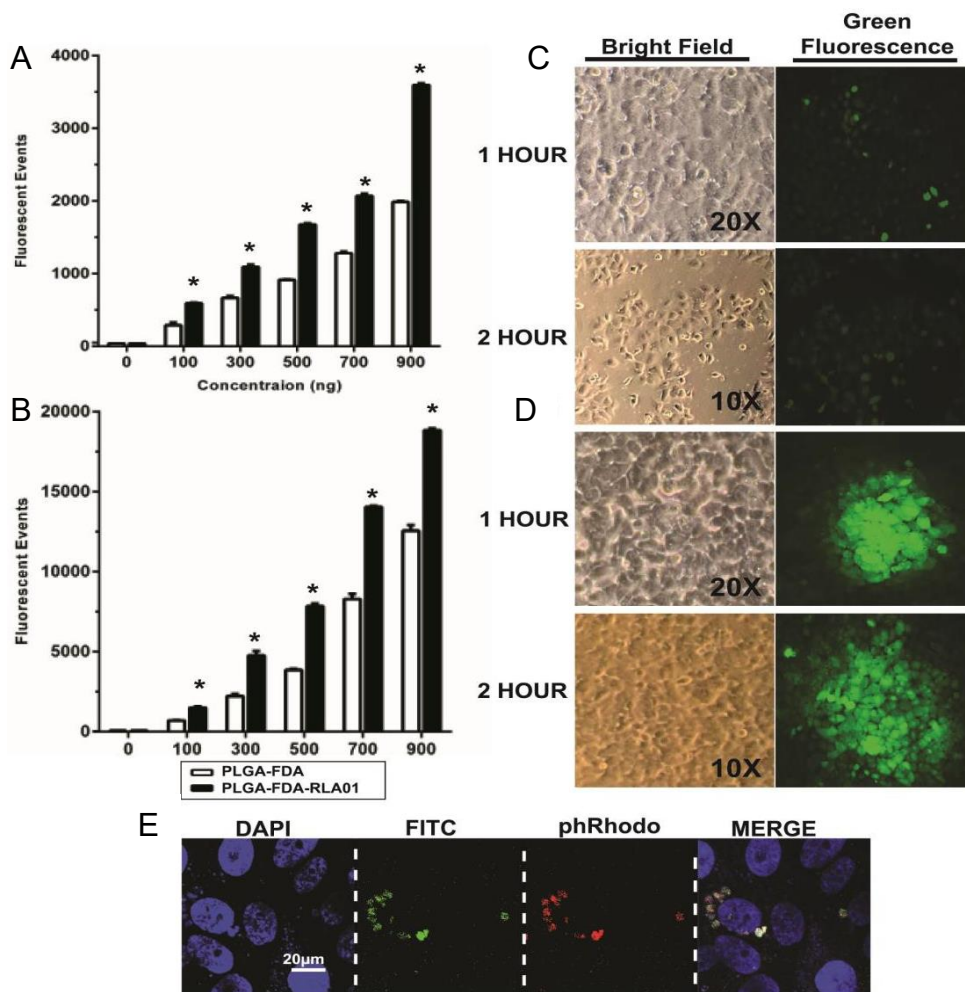

Figure 3

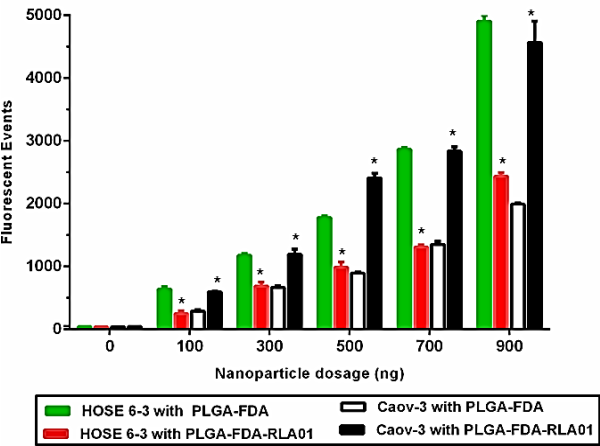

Figure 4

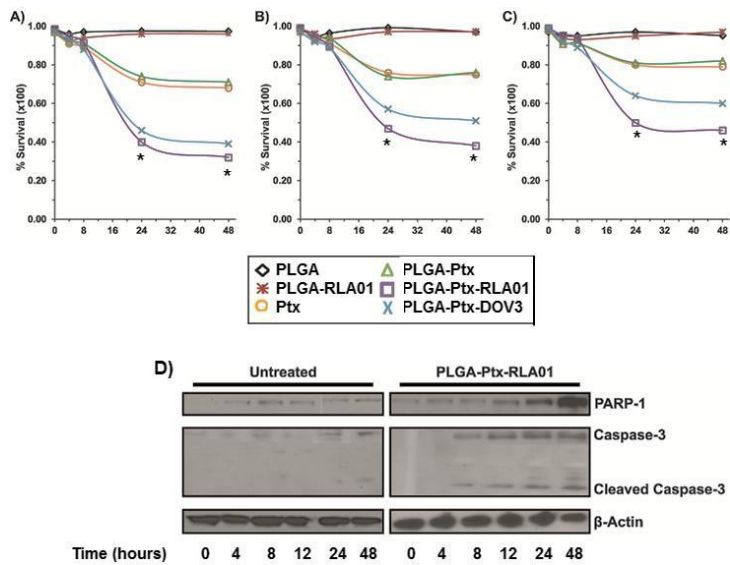

Figure 5

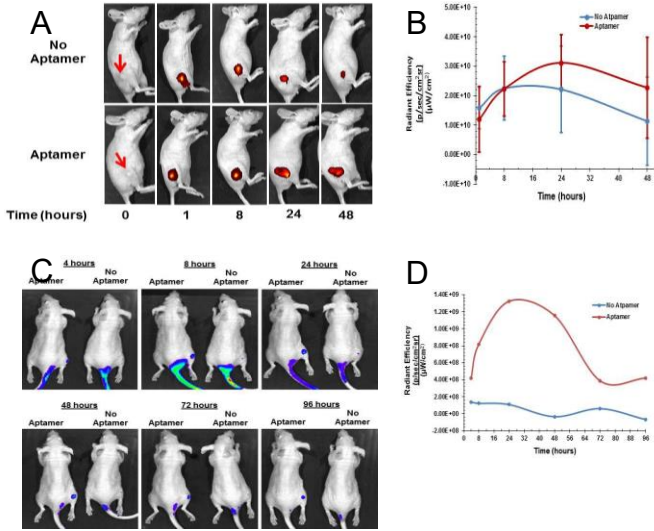

Supplement: Supplementary file 1 [file biomolecules-15-01123-s001.zip › biomolecules-3761562-original-images.pdf]
